# Kefir Peptides Prevent Estrogen Deficiency-Induced Bone Loss and Modulate the Structure of the Gut Microbiota in Ovariectomized Mice

**DOI:** 10.3390/nu12113432

**Published:** 2020-11-09

**Authors:** Min-Yu Tu, Kuei-Yang Han, Gary Ro-Lin Chang, Guan-Da Lai, Ku-Yi Chang, Chien-Fu Chen, Jen-Chieh Lai, Chung-Yu Lai, Hsiao-Ling Chen, Chuan-Mu Chen

**Affiliations:** 1 Department of Life Sciences, and Ph.D. Program in Translational Medicine, National Chung Hsing University, Taichung 402, Taiwan; du0807@yahoo.com.tw (M.-Y.T.); gary590422@yahoo.com.tw (G.R.-L.C.); gtrskyliner34@hotmail.com (G.-D.L.); balance1981@gmail.com (C.-F.C.); shoulia2001@yahoo.com.tw (J.-C.L.); 2 Aviation Physiology Research Laboratory, Kaohsiung Armed Forces General Hospital Gangshan Branch, Kaohsiung 820, Taiwan; 3 Department of Health Business Administration, Meiho University, Pingtung 912, Taiwan; 4 Department of Biomedical Engineering, Hungkuang University, Taichung 433, Taiwan; 5 Department of Family Medicine, Jen-Ai Hospital, Dali Branch, Taichung 402, Taiwan; shinchen@livemail.tw (K.-Y.H.); smallhead1230@yahoo.com.tw (K.-Y.C.); 6 Department of Orthopedic Surgery, Jen-Ai Hospital, Dali Branch, Taichung 402, Taiwan; 7 Department of Orthopedic Surgery, Taichung Armed Forces General Hospital, Taichung 411, Taiwan; 8 Graduate Institute of Aerospace and Undersea Medicine, National Defense Medical Center, Taipei 114, Taiwan; multi0912@gmail.com; 9 Department of Biomedical Sciences, Da-Yeh University, Changhua 515, Taiwan; bellchen@mail.dyu.edu.tw; 10 Department of Bioresources, Da-Yeh University, Changhua 515, Taiwan; 11 The iEGG and Animal Biotechnology Center, National Chung Hsing University, Taichung 402, Taiwan; 12 Rong Hsing Research Center for Translational Medicine, National Chung Hsing University, Taichung 402, Taiwan

**Keywords:** kefir peptides, dairy milk protein, osteoporosis, ovariectomized (OVX) mice, 16S rDNA, gut microbiota

## Abstract

Osteoporosis is a major skeletal disease associated with estrogen deficiency in postmenopausal women. Kefir-fermented peptides (KPs) are bioactive peptides with health-promoting benefits that are produced from the degradation of dairy milk proteins by the probiotic microflora in kefir grains. This study aimed to evaluate the effects of KPs on osteoporosis prevention and the modulation of the composition of the gut microbiota in ovariectomized (OVX) mice. OVX mice receiving an 8-week oral gavage of 100 mg of KPs and 100 mg of KPs + 10 mg Ca exhibited lower trabecular separation (Tb. Sp), and higher bone mineral density (BMD), trabecular number (Tb. N) and bone volume (BV/TV), than OVX groups receiving Ca alone and untreated mice, and these effects were also reflected in bones with better mechanical properties of strength and fracture toughness. The gut microbiota of the cecal contents was examined by 16S rDNA amplicon sequencing. α-Diversity analysis indicated that the gut microbiota of OVX mice was enriched more than that of sham mice, but the diversity was not changed significantly. Treatment with KPs caused increased microbiota richness and diversity in OVX mice compared with those in sham mice. The microbiota composition changed markedly in OVX mice compared with that in sham mice. Following the oral administration of KPs for 8 weeks, the abundances of *Alloprevotella*, *Anaerostipes*, *Parasutterella*, *Romboutsia*, *Ruminococcus_1* and *Streptococcus* genera were restored to levels close to those in the sham group. However, the correlation of these bacterial populations with bone metabolism needs further investigation. Taken together, KPs prevent menopausal osteoporosis and mildly modulate the structure of the gut microbiota in OVX mice.

## 1. Introduction

Osteoporosis, which is characterized by low bone mass and the disruption of bone structure, is a major public health concern in postmenopausal women [1]. According to statistical data from the International Osteoporosis Foundation, osteoporotic fractures occur in one in three women worldwide older than 50 years during their lifetime. These fractures are usually accompanied by pain, disability and an increased mortality rate. In addition to the impact on health, osteoporosis also causes a huge economic burden. The cost is expected to increase to USD 25.3 billion in the US by 2025 [2]. Therefore, the prevention of osteoporosis becomes a critical issue in order to decrease the economic burden of managing osteoporosis and improving the life quality of patients.

Many studies have focused on finding safe, cost-effective and natural approaches to treat osteoporosis without side effects. Among these, the consumption of milk, yogurt and other fermented dairy products should be a good choice, because they are widely available and are considered a healthy lifestyle in many countries. These products are excellent sources of bioactive proteins, vitamins and minerals, as well as prebiotics or probiotics, with a range of health benefits, including bone health [3]. Kefir is a fermented milk similar to yogurt with a history of over one hundred years. It was first consumed in Russia and European countries, and became popular in Asia in recent years. Accumulating reports have indicated that the consumption of kefir is associated with many health benefits [4,5]. These benefits partly originate from the functions of some kefir peptides (KPs), which are produced during fermentation via the degradation of milk proteins by the microorganisms in kefir grains. The release and peptide profile of KPs are influenced by the different fermentation conditions and microbial communities of kefir grains [6]. To date, only a few peptides, mainly from the casein protein, were characterized with anti-hypertension, anti-microorganism, immune-modulation and opioid properties [7]. Most of the bioactive KPs remain to be identified for their functions.

The gut microbiota generally refers to the group of microorganisms that inhabit the intestines with symbiotic, commensal or pathogenic relationships with their hosts. Numerous studies have demonstrated that the gut microbiota can shape many aspects of host physiological processes, including metabolic functions, nutrient absorption, immune responses and hormone secretion. Because bone homeostasis is affected by metabolic pathways, immune systems and the hormone environment, the gut microbiota can also influence the bone metabolism balance via these pathways [8]. Most studies have reported that the modulation of the gut microbiota using probiotics, such as *Lactobacillus spp.* [9,10,11,12,13], or the products of the degradation of prebiotics, such as short-chain fatty acid [13,14], can increase bone BMD and promote bone formation, indicating that the consumption of dairy products may lead to a higher peak bone mass [8].

Few studies have explored the efficacy of KPs in the prevention of estrogen-associated bone loss [15,16]. The consumption of kefir or kefir-like dairy products may have a great impact on the structure of the gut microbiota [17,18], but the link between gut microbiota changes and bone health is limited. Thus, we used an ovariectomized (OVX) murine model to simulate estrogen-associated bone loss, and then fed the mice with KPs for 8 weeks. Afterward, the femoral bones were removed to examine the bone mass and bone structure, and the feces in the cecal segment of the large intestine was collected to analyze the gut microbiota.

## 2. Materials and Methods

### 2.1. Kefir Peptides (KPs) Preparation

KP powder (KEFPEP) was purchased from Phermpep Biotech Co. (Taichung, Taiwan). The kefir starter grains were firstly inoculated (5%, w/v) in sterilized goat milk at 20 °C for 20 h for activation. The grains were then retrieved through a sieve and then were re-inoculated (10%, w/v) in fresh sterilized goat milk at 20 °C for 20 h. After the grains were filtered, the fermented supernatant products were spray-dried into KP powder as described previously [19,20,21]. The peptide content was determined as triglycine equivalents in g per 100 g sample by the O-phthalaldehyde (OPA) method [19], which was 23.1 g/100 g within the sample. The commercial KEFPEP powder contained 23.1% peptides, 26.1% fat, ~50% carbohydrates, 0.28% sodium, and ~3% calcium [21].

### 2.2. Animal Experiments

Thirty 8-week-old female C57BL/6J mice were obtained from the National Laboratory Animal Center (Taipei, Taiwan). The mice were housed in an air-conditioned room (22 ± 2 °C/50 ± 10% humidity) with an automatic 12 h light-dark cycle, and were allowed free access to a regular rodent diet (Altromin^®^ 1324 FORTI, Altromin GmbH, Lage, Germany) and water throughout the experiment. At 16 weeks of age, bilateral ovariectomy (24 mice) or sham surgery (6 mice) was performed. Two weeks later, the OVX mice were randomly divided into four groups (each *n* = 6) and treated as follows: (1) water/OVX, (2) Ca/OVX (10 mg of CaCO3 per kilogram of body weight), (3) KPs/OVX (100 mg of KPs per kilogram of body weight) and (4) KPs + Ca/OVX (100 mg of KPs + 10 mg of CaCO3 per kilogram of body weight). All the mice were subjected to daily oral gavages (0.1 mL) for 8 weeks. The body weight of the mice was recorded weekly. At the end of treatment, all the mice were sacrificed and their femoral bones were removed. The bones were immersed immediately in the fixation solution (4% formaldehyde in phosphate-buffered saline (PBS)) for one day and then were rinsed with PBS for subsequent characterization.

The cecal segments of the large intestines from the sham, OVX and KPs/OVX groups (each *n* = 3) were removed with their contents, placed immediately in liquid nitrogen, and then stored at −80 °C until ready for analysis. The present animal study was approved by the Institutional Animal Care and Use Committee (IACUC) of National Chung Hsing University (Taichung, Taiwan) with the IACUC No. 104-095.

### 2.3. Microcomputed Tomography (μ-CT)

The bone mineral density and trabecular microstructure of the right femur were examined using a high-resolution μ-CT scanner (Skyscan 1076 system; Bruker, Kontich, Belgium) at a resolution of 9 μm. The resulting image files were imported into CTAn software (Skyscan; Bruker, Kontich, Belgium) for three-dimensional (3D) image generation and the measurement of morphometric parameters, including the bone mineral density (BMD) and trabecular bone volume (BV/TV), thickness (Tb. Th), number (Tb. N) and separation (Tb. Sp). The structures of the trabecular bones were reconstituted using 100 μ-CT slices, which were approximately 0.9 mm in thickness from the growth plate of the distal femur.

### 2.4. Nanoindentation

The mechanical properties of the femur cortical bone were analyzed using a nanoindenter (TriboLab, Hysitron Inc., Minneapolis, MN, USA). A Berkovich diamond indenter with a tip radius of 50 nm was used to indent the polished surfaces of the cortical bone from the outer side to the inner side (near the bone marrow). For each sample, at least three series of indentation tests across the cortical bone shell (thickness of approximately 130–160 µm) were performed. The measurements obtained for the three parts of the residual indentation area of the cortical bone were averaged. The hardness and elastic modulus of the cortical bone at different locations were then calculated according to the indentation load–depth curves and Oliver–Pharr relationship, as described previously. To observe the fracture and estimate the fracture resistance, indentations were applied to the cortical bone surfaces under a high load of 500 mN. Scanning electron microscopy (SEM) was used to observe the fracture around the indented regions, and the residual indentation area was calculated according to the indentation load–depth curves.

### 2.5. Scanning Electron Microscopy

The distal femurs were trimmed in the sagittal plane and treated with a 5% sodium hypochlorite solution to expose the trabecular bone. All the skeletal samples were treated by dehydrating with acetone, air-drying, mounting on stubs, and coating with gold/palladium using an ion sputter (Hitachi E101, Tokyo, Japan), followed by examination using a scanning electron microscope (FEI Inspect S, Hillsboro, OR, USA).

### 2.6. Gut Microbiota

The collected cecal contents were submitted for gut microbiota analysis. From the DNA sampling to the final data, including nucleic acid extraction, PCR amplification, product purification, 16S rDNA amplicon sequencing and subsequent bioinformatics, the analyses were conducted by the Biotools Microbiome Research Center (Taipei, Taiwan). To guarantee the reliability of the data, quality control was performed at each step of the procedure. Briefly, the total genomic DNA from the samples was extracted using a QIAamp PowerFecal DNA Kit (Qiagen, Redwood, CA, USA). The DNA concentration was determined and adjusted to 5 ng/µL for PCR amplification. A specific primer set (319F: 5′-CCTACGGGNGGCWGCAG-3′ and 806R: 5′- GACTACHVGGGTATCTAATCC -3′) was used for PCR amplification of the V3-V4 region of the 16S rRNA gene. The PCR reaction was carried out with KAPA HiFi HotStart ReadyMix (Roche, Basel, Switzerland) under the following PCR conditions: 95 °C for 3 min; 25 cycles of 95 °C for 30 s, 55 °C for 30 s, 72 °C for 30 s; 72 °C for 5 min and hold at 4 °C. The PCR products were monitored on 1.5% agarose gel. Samples with a bright main strip around 500 bp were chosen and purified using the AMPure XP beads (Beckman Coulter, Indianapolis, IN, USA). A secondary PCR was performed by using the 16S rRNA V3-V4 region PCR amplicon and Nextera XT Index Kit with dual indices and Illumina sequencing adapters (Illumina, San Diego, CA, USA). The indexed PCR product quality was assessed on the Qubit 4.0 Fluorometer (Thermo Scientific, Alvarado, TX, USA) and Qsep100^TM^ system (Bioptic Inc., La Canada Flintridge, CA, USA). Equal amounts of the indexed PCR product were mixed to generate the sequencing library. At last, the library was sequenced on an Illumina MiSeq platform and paired 300 bp reads were generated.

### 2.7. Statistical Analysis

All the data were presented as means ± SEM (standard error of the mean) or means ± SD (standard deviations). Multiple group comparisons were performed using one-way ANOVA and Duncan’s post hoc test, and the statistical significances (*p* < 0.05) were indicated by different letters. The relative abundances of the gut microbiota were compared by one-way ANOVA and Tukey’s post hoc test, and the statistical significances were indicated by asterisks.

## 3. Results

### 3.1. Body Weight and Affected Organ Weight

**Figure 1:**
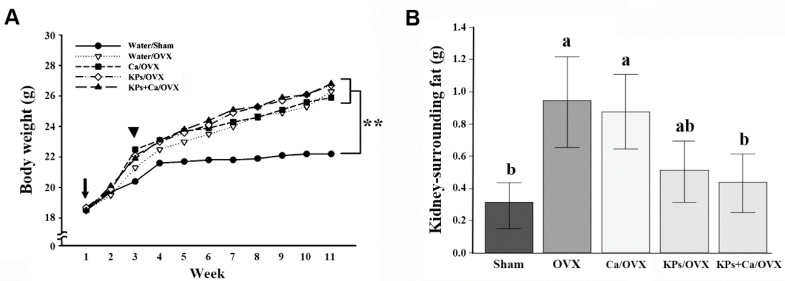
Body weight and kidney-surrounding fat. (**A**) The body weight was measured weekly from the onset of ovariectomy surgery to the end of the experiment. Water/Sham group: blank surgery mice fed with ddH_2_O, Water/OVX group: ovariectomy surgery mice fed with ddH_2_O; Ca/OVX group: ovariectomy surgery mice fed with 10 mg/kg CaCO3; KPs/OVX group: ovariectomy surgery mice fed with 100 mg/kg kefir peptides; KPs+Ca/OVX group: ovariectomy surgery mice fed with 100 mg/kg kefir peptides + 10 mg/kg CaCO3. In this panel, the arrow indicates the onset of surgery and the arrowhead indicates the beginning of oral administration. The mean body weights were higher in all the OVX groups than in the sham group (**, *p* < 0.01). (**B**) The kidney-surrounding fat was removed and measured immediately at the end of the experiment. The data are presented as means ± SEM (*n* = 6). Statistical analysis was performed by one-way ANOVA and Duncan’s post hoc test, and statistical significances are indicated by different letters (a, b; *p* < 0.05).

The gain rates of the body weight were different between the OVX and the sham mice (Figure 1A). At the end of the study, the body weights of the OVX groups were on average higher than those of the sham group (*p* < 0.01). Differences were also observed in the deposits of kidney-surrounding fat. Due to ovariectomy, the untreated OVX mice accumulated more kidney-surrounding fat than the sham mice (*p* < 0.05); however, the treatments with KPs and KPs + Ca (*p* < 0.05) reduced the kidney-surrounding fat. By contrast, treatment with Ca alone showed little or no effect on the reduction of the fat deposits around the kidneys (Figure 1B).

### 3.2. Effects of KPs on BMD and Bone Structure

**Figure 2:**
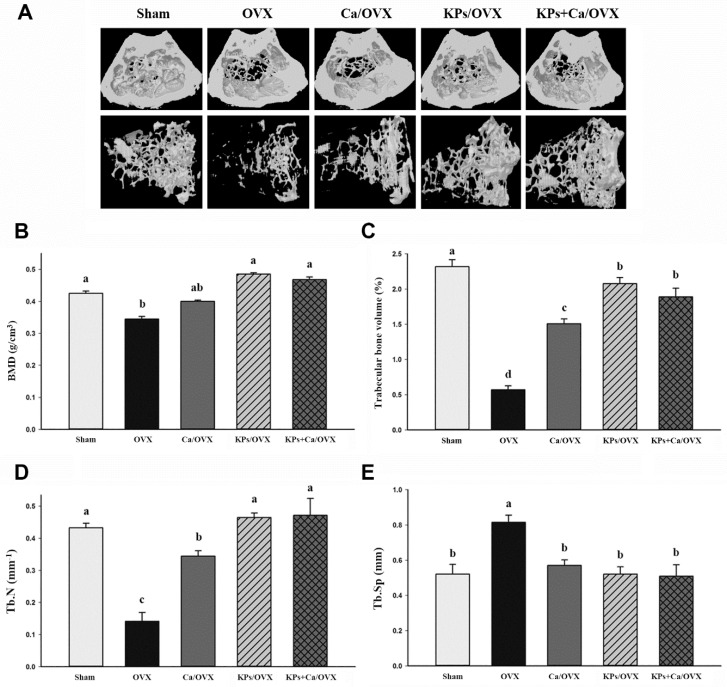
Micro-CT analysis of the femur. (**A**) Three-dimensional images of the distal femur. The images in the upper panel show the transverse section of the femur, and images in the lower panel show the structure of the trabecular bone. (**B**–**E**) show the morphological parameters of μ-CT analysis, including bone mineral density (**B**), trabecular bone volume (Tb. BV/TV) (**C**), trabecular number (Tb. N) (**D**) and trabecular separation (Tb. Sp) (**E**). The data are presented as means ± SEM (*n* = 6). Statistical analysis was performed by one-way ANOVA and Duncan’s post hoc test, and statistical significances are indicated by different letters (a, b, c; *p* < 0.05).

The effects of KPs on BMD and bone structure were examined by μ-CT. The OVX mice without treatment showed severe losses of trabecular bones, and mild attenuations in the thickness of the cortical bones, compared with the sham mice (Figure 2A). Although the administration of Ca alone conferred a slight protection from large-scale bone loss, larger spaces or a less-dense structure still appeared in the trabecular bones in the OVX mice receiving Ca alone. The OVX mice receiving the treatments of KPs and KPs + Ca exhibited trabecular bones with a healthy or dense appearance compared with the sham mice, suggesting that KPs or KPs combined with Ca can provide better protection than Ca alone.

The protection of KPs is reflected in the parametric changes of trabecular bones. The BMD of the untreated OVX group (0.34 g/cm^3^) showed about a 19% reduction compared with the sham group (0.42 g/cm^3^) (*p* < 0.05). The BMD values were 0.40, 0.48 and 0.47 g/cm^3^ in the OVX groups following treatments with Ca, KPs and KPs + Ca, respectively (Figure 2B). Ovariectomy caused a relatively low trabecular bone volume (Tb. BV/TV) in the untreated OVX group (0.57%), showing a ~75% reduction compared with that in the sham group (2.32%) (*p* < 0.001). The bone volumes increased to 1.51%, 2.08% and 1.87% in the OVX groups receiving Ca, KPs and KPs + Ca, respectively (Figure 2C). The untreated OVX mice (0.14/mm^3^) also showed a relatively lower trabecular bone number (Tb. N), with a ~67% reduction compared with the sham group (0.43/mm^3^) (*p* < 0.01). However, following treatments with Ca, KPs and KPs + Ca, the Tb. N increased to 0.34, 0.47 and 0.47/mm^3^, respectively, among which the groups treated with KPs and KPs + Ca were comparable to the sham group (Figure 2D). By contrast, the trabecular separation (Tb. Sp) increased in the untreated OVX mice (0.82 mm), showing a ~58% increase compared with the sham group (0.52 mm) (*p* < 0.05). However, following treatments with Ca, KPs and KPs + Ca, the Tb. Sp decreased to 0.57, 0.52 and 0.51 mm, respectively, in which the groups treated with KPs and KPs + Ca were comparable to the sham group (*p* > 0.05) (Figure 2E).

### 3.3. Effect of KPs on the Mechanical Indices of the Cortical Bones

**Figure 3:**
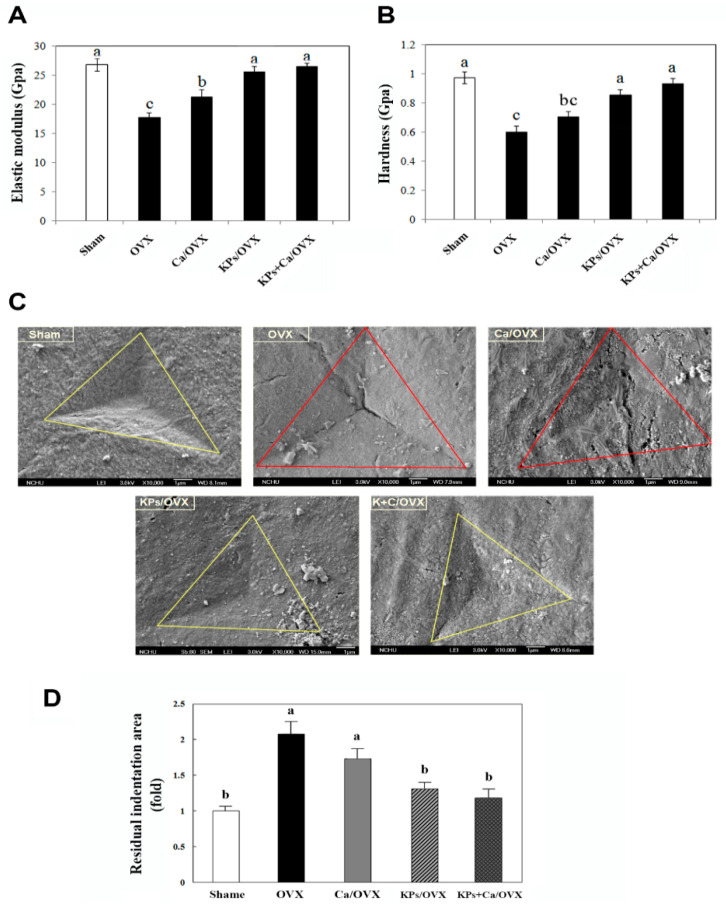
Nanoindentation analysis of the mechanical properties of cortical femoral bones at the end of oral administration. Each sample was analyzed by at least three series of outer-to-inner indentation tests (15 points) across the transverse section of the cortical bone shell (130–160 µm in thickness). The mechanical properties of the elastic modulus (**A**) and hardness (**B**) were compared among the groups. (**C**) The indented surfaces were further examined by scanning electron microscopy. The residual nanoindentation areas are marked by triangles and were quantitated using ImageJ software. The relative residual nanoindentation areas were calculated by comparison with the sham group (**D**). The data are presented as means ± SEM (*n* = 6). Statistical analysis was performed by one-way ANOVA and Duncan’s post hoc test, and statistical significances are indicated by different letters (*p* < 0.05).

The mechanical indices (elastic modulus and hardness) of the cortical bones were examined by nanoindentation. The cortical elastic modulus was significantly decreased in the OVX group (18.6 GPa), showing a ~32% reduction compared with that in the sham group (27.2 GPa) (Figure 3A; *p* < 0.01). The cortical elastic modulus values substantially increased to 22.5, 26.4 and 27.0 GPa after treatments with Ca, KPs and KPs + Ca, respectively, in which the groups treated with KPs and KPs + Ca were comparable to the sham group. A similar trend was also observed in the change of the cortical hardness. The cortical hardness markedly decreased in the OVX group (0.61 GPa), showing a ~36% reduction compared with that in the sham group (0.95 GPa) (Figure 3B; *p* < 0.01). Treatments with Ca, KPs and KPs + Ca conferred the OVX mice with higher cortical hardness values up to 0.73, 0.83 and 0.91 GPa, respectively, in which the groups treated with KPs and KPs + Ca were comparable to the sham group.

The indented surfaces of the cortical bones were examined by SEM, and the residual indentation areas, marked with the triangles in Figure 3C, were measured. The residual indentation area of the untreated OVX group was 2.1-fold higher than that of the sham group (Figure 3D; *p* < 0.05). A higher residual indentation area indicates poor bone strength and fracture toughness. Treatment with Ca alone (1.8-fold higher than sham) conferred an insignificant change in the residual indentation areas. However, treatments with KPs (1.3-fold higher than sham) and KFPs + Ca (1.2-fold higher than sham) significantly decreased the residual indentation areas (*p* < 0.05), and both were comparable with the sham group.

### 3.4. Effect of KPs on the Gut Microbiota

**Figure 4:**
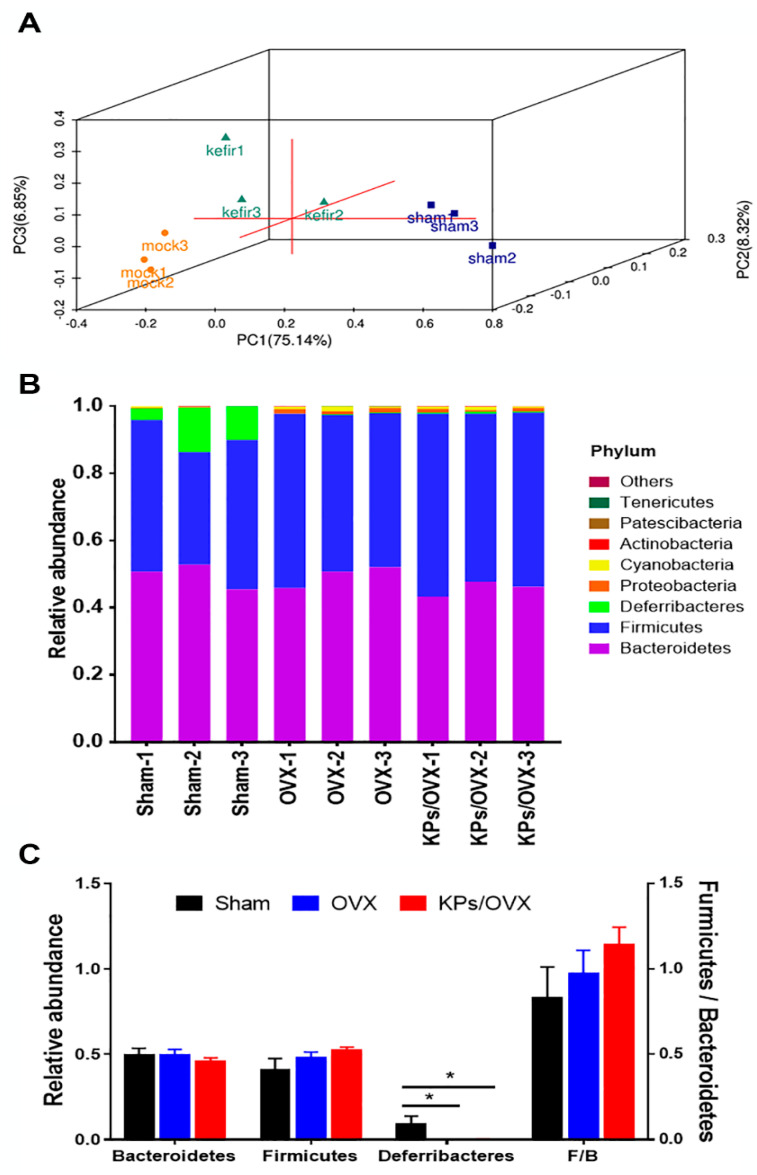
Analysis of the gut microbiota by 16S rDNA amplicon sequencing. (**A**) Principal co-ordinates analysis (PCoA), a common method of β-diversity index analysis, was used to evaluate the differential significance of the total abundance of the gut microbiota among the groups. PC1: Pricipal coordinate analysis axis 1; PC2: Pricipal coordinate analysis axis 2; PC3: Pricipal coordinate analysis axis 3. (**B**) Structural comparison of the gut microbiota at the phylum level. (**C**) Relative abundances of the phyla Bacteroidetes Firmicutes and Deferribacteres, and the ratio of Firmicutes to Bacteroidetes (F/B), a marker of obesity. Statistical analysis was performed by one-way ANOVA and Tukey’s post hoc test. Statistical significances are indicated by asterisks (*, *p* < 0.05).

The gut microbiota of the cecal contents collected from the sham, OVX and KPs/OVX groups were evaluated by 16S amplicon sequencing. In total, 316 operational taxonomic units (OTUs), equal to 30,582 sequences/sample, with >97% identity were used for analysis. First, the α- and β-diversity were calculated to evaluate the effect of ovariectomy and KP treatment on the total abundance of the gut microbiota. Principal co-ordinates analysis (PCoA) showed distinct clustering of the gut microbiota from the sham, OVX and KPs/OVX groups, but comparisons via the β-diversity indices of unweighted uniFrac and weighted uniFrac indicated that the difference was not significant (Figure 4A and Table 1). Alpha-diversity indices were calculated to evaluate the change in the microbiota richness and diversity between various groups. The OVX and KPs/OVX groups showed significantly higher observed OTU, Ace and Chao1 indices than the sham group (*p* < 0.05), the KPs/OVX group showed significantly higher Shannon and Simpson indices than the sham group (*p* < 0.05), and no significant differences in the α-diversity indices were observed between the OVX and KPs/OVX groups (*p* > 0.05) (Table 1).

**Figure 5:**
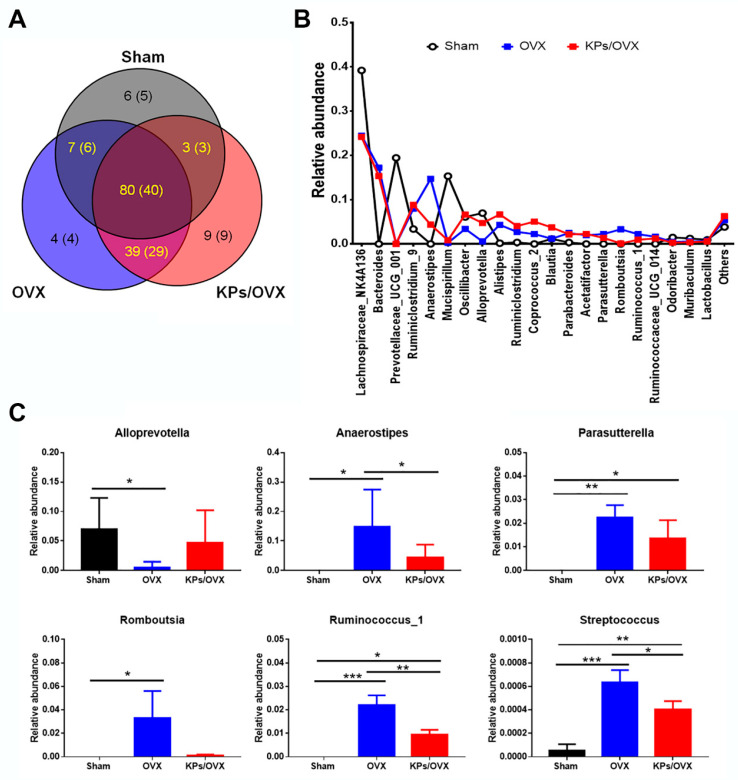
Structural comparison of the gut microbiota at the genus level. (**A**) Venn diagram comparing the observed operational taxonomic units in the gut microbiota of sham, OVX and KPs/OVX mice. (**B**) Major bacterial genera identified in this study. (**C**) Mean relative abundances of the genera *Alloprevotella*, *Anaerostipes*, *Parasutterella*, *Romboutsia*, *Ruminococcus_1* and *Streptococcus*. Compared with the sham group, these bacterial genera were inhibited or enriched in the OVX group but were reversed in the KPs/OVX group. The data are presented as means ± standard deviation (SD) (*n* = 3). Statistical analysis was performed by one-way ANOVA and Tukey’s post hoc test. Statistical significances are indicated by asterisks (*, *p* < 0.05; **, *p* < 0.01 and ***, *p* < 0.001).

Next, we compared the microbiota structure at different hierarchical levels. At the phylum level, the gut microbiota of mice mainly comprised Bacteroidetes, Firmicutes and Deferribacteres, but the OVX groups had a lower relative abundance (RA) of Deferribacteres than the sham group (Figure 4B). The Firmicutes to Bacteroidetes (F/B) ratio, a known marker of obesity [20], was slightly higher in the OVX groups than in the sham group. The oral administration of KPs increased the F/B ratio, but the differences in the F/B ratio among the sham, OVX and KPs/OVX groups were not statistically significant (Figure 4C; *p* > 0.05). To identify specific microbial populations affected by oral KPs treatment, the RA of the gut microbiota was compared at the genus level. In this study, 148 of 316 OTUs were annotated to the genus level, accounting for 73 genera. A Venn diagram was constructed to represent the relationship among the three groups. The quantity of OTUs (genera) unique to each group was less than 0.6% in abundance, while the genera shared by the sham, OVX and KPs/OVX groups were more than 99% in abundance (Figure 5A). The identified genera with >1% abundance are shown in Figure 5B; the other genera accounted for a very small fraction of the total abundance. At the genus level, the gut microbiota of the sham group comprised Lachnospiraceae_NK4A136, Prevotellaceae_UCG_001, Mucispirillum, Oscillibacter and Alloprevotella predominantly (87% abundance in total); however, the abundance of the predominant genera decreased significantly in the OVX group (29% abundance). By contrast, the sham group contained relatively lower abundances of Bacteroides, Ruminiclostridium_9, Anaerostipes, Alistipes, Ruminiclostridium, Coprococcus_2, Parabacteroides, Acetatifactor, Parasutterella, Romboutsia, Ruminococcus_1 and Ruminococcaceae_UCG014 (<5% abundance); however, these genera were enriched importantly in the OVX group (63% abundance). Thus, the structure of the gut microbiota was markedly affected by ovariectomy. Moreover, we were interested in the bacterial genera affected by KPs treatment. The oral administration of KPs for 8 weeks in OVX mice revealed that the abundances of the genera Alloprevotella, Anaerostipes, Parasutterella, Romboutsia, Ruminococcus_1 and Streptococcus were restored to a level close to those in the sham group (Figure 5C). Among these bacterial populations, the abundances of the genera Anaerostipes, Ruminococcus_1 and Streptococcus in the KPs/OVX group were significantly different compared with those in the OVX group (*p* < 0.05).

## 4. Discussion

Osteoporosis is a prevalent bone disease in the population of postmenopausal women. A safe and cost-effective natural product for osteoporosis prevention and treatment will help to decrease the incidence of osteoporosis in postmenopausal women. Kefir, fermented milk produced from kefir grains, has attracted widespread interest in the scientific community because it contains many bioactive peptides with health-promoting benefits. The association of kefir with osteoporosis was first published in our previous report [15], which demonstrated the benefits of kefir in preventing postmenopausal bone loss using an OVX rat model. Subsequently, we demonstrated the beneficial effects of kefir on bone mineral density and bone metabolism in a clinical trial of osteoporotic patients [16]. In this study, we further demonstrated the function of kefir peptides (KPs) on osteoporosis prophylaxis, and provided more evidence to gain insight into the association of KPs with the structural change of the gut microbiota in OVX mice.

OVX rats or mice are the widely used animal models of postmenopausal osteoporosis. The remarkable elevation of the body weight and kidney-surrounding fat in the untreated OVX mice indicated the success of the present animal model. At the end of this study, we found that the kidney-surrounding fat decreased in OVX mice with KPs treatment, suggesting the modulating activities of KPs on lipid metabolism. The functions of kefir or KPs in inhibiting lipogenesis were fully discussed in our previous reports [19,21,22,23,24,25].

Calcium carbonate and other calcium supplements are often used as an aid to prevent osteoporosis and to treat patients, along with the other medications. The present bone analysis indicated that supplementation with calcium carbonate alone provided little protection against OVX-induced bone loss, but greater protection followed KPs treatment and the combined use of KPs and calcium carbonate. The protection from OVX-induced bone loss also conferred the bones with higher mechanical strength to resist external pressure. Thus, the administration of KPs and KPs + Ca maintained the mechanical parameters of elastic moduli and hardness at a level similar to that in the sham group, with a marked difference compared with that in the untreated OVX mice or mice treated with Ca alone. The OVX mice treated with KPs or KPs + Ca also exhibited a lower residual indentation area than untreated OVX mice, suggesting the higher strength to resist bone fracture (Figure 3D). The microarchitectural and mechanical changes in the bones in OVX mice were consistent with those observed in our previous OVX rat model with different dosages of KPs [15]. In addition to structural and mechanical properties, the bone-protective effects of KPs can also be monitored by serum bone turnover markers, such as alkaline phosphatase (ALP) and C-terminal telopeptide fragment of type I collagen C-terminus (CTX-1). In this study, we did not evaluate the effects of KPs on serum bone turnover markers, but a reduced serum level of ALP and CTX-1 has been found in OVX rats treated with KPs. These results confirmed the outstanding effect of KPs in preventing postmenopausal osteoporosis. The recent studies using proteomic or peptidomic approaches to analyze kefir beverages have provided us with a comprehensive understanding of the released peptide composition during the fermentation process [6,7,26,27,28]. Hundreds to thousands of peptides have been identified in those studies, but only a few contained 100% sequence homology to known functional peptides [6,7,28]. Most KPs have not been investigated fully, and many are released as a precursor form of the functional peptide, in which the functional sequence may be contained within the released peptide [6,26,27,28]. Currently, the KPs with osteo-protective potential remain unclear. However, various studies have indicated the positive effect of bioactive peptides derived from casein and whey proteins (two major proteins of milk) on bone metabolism, a finding that may provide directions to understand the effects of active components of KPs on bone metabolism. Casein phosphopeptides (CPPs), a family of casein-derived peptides containing serine phosphate able to bind and solubilize calcium, may promote calcium absorption by the intestine and increase the bioavailability of calcium ions by other tissues in the body. There are two reports that have indicated the osteo-protective effect of CPPs in rats due to the increased calcium absorption caused by the administration of CPPs orally, or by supplementing CPPs in the rodent diet [29,30]. KPs were also shown to promote calcium absorption in Caco-2 cells in our previous study [15]. In the study of Ebner et al. [6], some identified peptides in bovine milk fermented with kefir grains showed similar sequence coverages of caseins with known CPPs, e.g., αs1-casein (59–79), αs1-casein (43–58), β-casein (1–25) and β-casein (33–48) [31]. However, the positive correlation of CPPs with the promotion of calcium absorption remains debatable, because some studies have reported the opposite results [32,33]. As such, further investigations are required to evaluate the functions of CPPs in calcium absorption and their correlation with bone metabolism. Casein-derived antioxidative peptide (β-casein (185–191): VLPVPQK), whey derived-antioxidative (β-lactoglobulin (60–64): YVEEL) and angiotensin-converting enzyme (ACE) inhibitory (β-lactoglobulin (120–123): YLLF) peptides have also been shown to exert osteo-protective properties under in vitro or in vivo conditions [34,35,36,37]. The osteo-protective properties of these antioxidative and ACE-inhibitory peptides originate from their ability to suppress the inflammatory status and RAS (renin-angiotensin system) activity, respectively. Peptides homologous to the sequence of known antioxidative or ACE-inhibitory peptides were also identified in the abovementioned proteomic studies. Again, most of their functions remain to be further investigated. We have isolated and identified several peptide candidates responsible for bone protection using our materials, and their effects on osteoclastogenesis and osteoblastogenesis are currently being studied.

Following 8 weeks of treatment, we used high-throughput sequencing of bacterial 16S rDNA to compare the compositions of the gut microbiota in different groups (Figure 4 and Figure 5). According to α-diversity analysis, we found that the OVX group had significantly higher Chao1 and Ace indices than the sham group (Table 1). The Chao1 and Ace indices are usually used to indicate the richness of the gut microbiota; thus, our findings indicated that ovariectomy increased the richness of gut microbiota. Additionally, the Shannon and Simpson indices, which are used to represent the diversity of microbiota, were not significantly changed between the OVX and sham groups, suggesting the diversity of the gut microbiota was not affected significantly after ovariectomy (Table 1). Eight-week administration with KPs did not alter the α-diversity indices significantly compared with the OVX mice receiving mock treatment, indicating that the microbiota richness and diversity in OVX mice were not affected significantly by KPs treatment. However, significant differences were observed in the richness and diversity of the gut microbiota between the KPs/OVX and sham groups. The structure of the gut microbiota was changed markedly by ovariectomy, as indicated by the predominant bacterial populations being inhibited and many less-abundant bacterial populations being enriched at different hierarchical levels. These effects should be reasonable because estrogen plays important roles in the regulation of various metabolic pathways in women, particularly regarding its association with postmenopausal bone homeostasis [38]. At the cellular level, the central mechanism of estrogen deficiency-induced bone loss occurs via the promotion of osteoclast formation and the expansion of RANKL- and TNF-expressing cells [39,40]. The gut microbiota regulates bone homeostasis by influencing host metabolism, calcium absorption, the immune system and the endocrine system [8]. The interplay between estrogen and the gut microbiota is therefore apparent [41]. For example, an increased Firmicutes to Bacteroidetes (F/B) ratio in the gut microbiota has even been reported in OVX rats or mice [42,43,44]. In the present study, we observed a higher F/B ratio resulting from a mild increase in Firmicutes in both the OVX and KPs/OVX groups, although the differences did not reach a significant level. Consistent with the higher weight gain observed in all OVX mice (Figure 1A), our data supported using the F/B ratio as a microbiota marker of obesity [22]. The F/B ratio seemed to be higher in OVX mice with KPs treatment than sham mice, but the difference was not statistically important. At the phylum level, Deferribacteres exhibited decreased abundances in all OVX mice (Figure 4B,C). In healthy mice, Deferribacteres emerged as the third dominant phylum in the gut microbiota, and metagenomic and metatranscriptomic analysis revealed that the genes for cofactor, vitamin metabolism and amino acid metabolism were upregulated in the Deferribacteres family [45]. To further study the response of the gut microbiota to ovariectomy surgery and KPs treatment, the bacterial populations of all three groups were compared at the genus level. The predominant bacterial genera in the sham group included *Lachnospiraceae_NK4A136*, *Prevotellaceae_UCG_001*, *Mucispirillum*, *Oscillibacter* and *Alloprevotella*, which were decreased significantly in OVX mice (87% for sham vs. 29% for OVX). The minor populations *Bacteroides*, *Ruminiclostridium_9*, *Anaerostipes, Alistipes*, *Ruminiclostridium*, *Coprococcus_2*, *Parabacteroides*, *Acetatifactor*, *Parasutterella*, *Romboutsia*, *Ruminococcus_1* and *Ruminococcaceae_UCG_014* were significantly enriched in the OVX group (5% for sham vs. 63% for OVX), indicating that ovariectomy had a substantial effect on the alteration of the gut microbiota. At the genus level, the oral administration of KPs restored the abundances of six bacterial populations in OVX mice to a level close to that in sham mice. These genera were *Alloprevotella*, *Anaerostipes*, *Parasutterella*, *Romboutsia*, *Ruminococcus_1* and *Streptococcus* (Figure 5C). *Alloprevotella* was reported to be negatively correlated with nonalcoholic fatty acid liver and lipid accumulation [46]. The increased abundance of *Alloprevotella* may explain the reduction in kidney-surrounding fat accumulation in OVX mice receiving KP treatment (Figure 1B). Notably, the oral administration of KPs prevented nonalcoholic fatty acid liver and hyperlipidemia and obesity in our previous murine models [21,24]. *Parasutterella* and *Streptococcus* are potential harmful bacteria that were reported to be correlated with inflammatory bowel disease [47] and the progression of some tumors or cancers [48,49]. Additionally, *Streptococcus* has been reported to be positively correlated with body mass index in obese individuals [50]. *Romboutsia* were also reported to be obesity-related bacteria. Interestingly, KPs can reverse the alteration of the gut microbiota in OVX mice by enriching beneficial bacteria and decreasing potentially harmful pathogens. *Anaerostipes* and *Ruminococcus_1* are short-chain fatty acid (SCFA)-producing anaerobic bacteria. SCFAs, such as butyrate and propionate, are essential bacterial metabolites from carbohydrates in the gut because they evoke anti-inflammatory effects in the intestinal mucosa and promote bone metabolism [14,51,52]. We hypothesized that ovariectomy would reduce the abundance of *Anaerostipes* and *Ruminococcus* genera, and then KP treatment would reverse it. However, an opposite trend was observed in our study. High systemic concentrations of SCFAs were reported to be toxic and caused adverse effects in the host, mostly arising from the enhanced permeability of the gut barrier to increase the serum level of SCFAs [13,53]. These data imply that the increment in butyrate-producing bacteria after estrogen deficiency may produce excessive SCFAs in the intestine. Recently, Ma et al. [54] reported the correlation of a gut microbiota change with bone turnover parameters. In that report, *Ruminococcus*, *Clostridium*, *Coprococcus* and *Robinsoniella* were shown to be positively correlated with osteoclastic indicators (CTX, Tb.Sp) in OVX rats, but *Bacteroides* and *Butyrivibrio* showed the opposite patterns, and were negatively correlated with loss of bone mass. It is difficult to consider that one bacterial population will be absolutely correlated with specific physiological metabolic pathways or diseases. A healthy condition should be based on a more balanced structure of the microbiota in the host. The gut microbiota plays a critical role in the regulation of bone homeostasis by secreting various bacterial metabolites (SCFAs and bile acids) into the intestinal lumen, and influencing host intestinal barrier permeability, the immune system, hormone secretion and the gut–brain axis. Therefore, further mechanistic studies are needed to verify the causal role of gut microbiota in estrogen deficiency-induced osteoporosis.

## 5. Conclusions

In this study, we used the OVX mouse model to confirm the potential role of KPs in preventing menopausal osteoporosis, and indicated that the osteo-protective function of KPs is independent of calcium supplementation. Furthermore, our results suggested that the oral administration of KPs alters the structure of the gut microbiota in OVX mice by enriching the abundance of beneficial bacteria and reducing the abundance of potential pathogens.

## Figures and Tables

**Table 1:** The mean α- and β-diversity indices and the *P* values for various group comparisons.

	α-Diversity Index					β-Diversity Index	
Group	Observed-OTU	Ace	Chao1	Shannon	Simpson	Unweighted uniFrac	Weighted uniFrac
Sham	160	177.0	180.6	4.938	0.932	0.199	0.038
OVX	217	233.1	232.9	5.657	0.965	0.080	0.034
KPs/OVX	230	244.1	248.3	5.790	0.968	0.327	0.042
Comparison	*p* value of α-diversity					*p* value of β-diversity	
Sham vs OVX	**0.004**	**0.002**	**0.006**	0.055	0.062	0.441	0.645
Sham vs KPs/OVX	**0.001**	**0.001**	**0.002**	**0.028**	**0.049**	0.395	0.761
OVX vs KPs/OVX	0.480	0.514	0.363	0.850	0.979	0.077	0.299

Statistical significances were analyzed by one-way ANOVA and Tukey’s post-hoc test, and the bold values represent *p* < 0.05.
